# Role of Leukotrienes on Protozoan and Helminth Infections

**DOI:** 10.1155/2012/595694

**Published:** 2012-04-10

**Authors:** Alexandre P. Rogerio, Fernanda F. Anibal

**Affiliations:** ^1^Laboratory of Experimental Immunopharmacology, Federal University of Triângulo Mineiro, Rua Vigário Carlos, 162. 38025-380 Uberaba, MG, Brazil; ^2^Department of Morphology and Pathology, Federal University of São Carlos, Rodovia Washington Luis, km 235 Caixa Postal 676, 13565-905 São Carlos, SP, Brazil

## Abstract

Leukotrienes (LTs), formed by the 5-lipoxygenase-(5-LO-) catalyzed oxidation of arachidonic acid, are lipid mediators that have potent proinflammatory activities. Pharmacologic or genetic inhibition of 5-LO biosynthesis in animals is associated with increased mortality and impaired clearance of bacteria, fungi, and parasites. LTs play a role in the control of helminth and protozoan infections by modulating the immune system and/or through direct cytotoxicity to parasites; however, LTs may also be associated with pathogenesis, such as in cerebral malaria and schistosomal granuloma. Interestingly, some proteins from the saliva of insect vectors that transmit protozoans and secreted protein from helminth could bind LTs and may consequently modulate the course of infection or pathogenesis. In addition, the decreased production of LTs in immunocompromised individuals might modulate the pathophysiology of helminth and protozoan infections. Herein, in this paper, we showed the immunomodulatory and pathogenic roles of LTs during the helminth and protozoan infections.

## 1. Introduction

Leukotrienes (LTs), first described by Samuelsson's group [1, 2], are a class of lipid mediators involved in several diseases but classically known for their effects on asthma and allergy. The generation of leukotrienes (LTs) is dependent upon the action of 5-lipoxygenase (5-LO) in association with membrane-bound 5-lipoxygenase-activating protein (FLAP) on arachidonic acid (AA). AA is derived through the action of cytosolic phospholipase A_2_ (cPLA_2_) and/or secreted phospholipase A_2_ (sPLA_2_) on membrane phospholipids [3]. LTA_4_, an unstable precursor of all leukotrienes, is quickly metabolized to one of the two different classes of LTs, LTB_4_ (by LTA_4_ hydrolase) or LTC_4_ (by LTC_4_ synthase) and its metabolites (LTD_4_ and LTE_4_) [4]. Collectively, LTC_4_, LTD_4_, and LTE_4_ were previously known as the slow-reacting substance of anaphylaxis (SR-A) and are currently termed the cysteinyl LTs (cysLTs) [3, 4]. The receptors for LTB_4_ (BTL1 and BTL2) and cysteinyl LTs (CysLT1 and CysLT2) are cell surface G protein-coupled receptors [3]. Additionally, some studies support the existence of other CysLT receptors [5, 6]. Some cells express both BTLs and cysLTs; however, the expression of these receptors differs in different cells types. In addition, these receptors are also expressed on peripheral blood leukocytes [7, 8]. LT receptors and 5-LO are expressed mainly in immune cells [6], and LTs play important roles in innate and adaptive immune responses and are involved in several inflammatory and infectious diseases [4, 9]. For example, cysLTs increase vascular permeability and edema, and LTB_4_ is involved in leukocyte chemotaxis, lysosomal enzyme secretion, neutrophil degranulation, adhesion molecule expression, defensins and nitric oxide (NO) production, phagocytosis, and other functions [9]. LTs are produced during the interaction of phagocytes and microorganisms *in vitro* and experimental infections *in vivo *[9]. Pharmacologic or genetic approaches to reduce or block the LT biosynthesis pathways decrease the phagocytic and antimicrobial activities against bacteria [10], fungi [11], and parasites [12, 13]. In addition, immunodeficient individuals, such as HIV patients, are characterized by low LT production [14], which has been associated with impaired immune responses and infection control. LTs play important roles in both Th1 and Th2 immune responses, which are involved in the defense against protozoan and helminth infections, respectively. In light of the current research on the role of LTs in infectious diseases, we have divided the current review into two sections focusing on (1) protozoan infection and (2) helminth infection.

## 2. Leukotrienes and Protozoan Infection

Each year, protozoan parasites infect many people worldwide, mainly in developing countries, causing serious health, political, social, and economic problems. The major protozoan parasites with clinical importance for human diseases are *Plasmodium *ssp, *Leishmania *ssp, *Trypanosoma cruzi*, *Toxoplasma gondii*, *Trichomonas vaginalis, *and *Entamoeba histolytica* [15–17]. The first three of these organisms are obligate intracellular protozoan parasites that are transmitted to vertebrate hosts by insect vectors. *T. gondii *is also an obligate intracellular protozoan parasite; however, its transmission to human hosts occurs by ingestion of raw or undercooked meat containing tissue cysts or food or water contaminated with oocysts. *T. vaginalis* and *E. histolytica* are extracellular protozoan parasites. *T. vaginalis is* transmitted sexually (trophozoites) and *E. histolytica* is transmitted through food and water contaminated with cysts [15–17]. Protective immunity against protozoans is mediated mainly by T helper 1 (Th1) responses which are characterized by the production of inflammatory cytokines, such as IL-12, which is required for the development of the Th1 immune response, and interferon gamma (IFN-*γ*) and tumor necrosis factor alpha (TNF-*α*), which activate macrophages to produce NO, which is involved in the control of parasite replication [16, 18–20].

**Figure 1:**
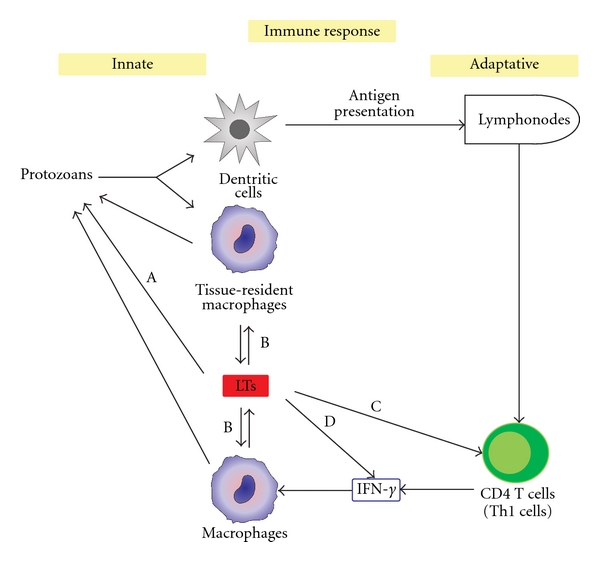
Modulation of innate and adaptive immune responses by LTs during the protozoan infections. Protective immunity against protozoans is mainly mediated by Th1 responses characterized by the production of inflammatory cytokines, such as IFN-*γ*, which activate macrophages to control of parasite replication. Macrophages are a source of LTs. (A) LTB_4_ induces cytotoxicity in parasites. (B) LTB_4_ and LTD_4_ favored parasite killing by macrophages. (C) LTB_4_ induces chemotaxis of CD4^+^ T cells. (D) LTB_4_ induces the production of Th1 cytokines, such as IFN-*γ*.

Reiner and Malemud [21, 22] conducted the first studies to demonstrate the role of leukotrienes in protozoan infection (*Leishmania* spp). Research in this area has increased in recent decades. The main effects of LTs, in both innate and adaptative immune responses, during the protozoan infections are illustrated in Figure 1. Mouse strains resistant (C57BL/6) to *Leishmania* infection mount Th1 immune responses against *Leishmania*. In contrast, infection of susceptible mouse strain (BALB/c) is associated with the development of a Th2 immune response. *In vitro *studies have demonstrated increased LTC_4_ production in splenocytes and macrophages from *L. donovani*-infected or uninfected BALB/c mice upon stimulation with nonspecific (phytohemagglutinin) or specific (*L. donovani* amastigotes) stimuli [21, 22]. In another study, splenocytes from BALB/c mice stimulated with antigens from *L. major* promastigotes displayed increased LTB_4_ and IL-4 production with concomitant decreases in IFN-*γ* and TNF-*α* production [23]. Serezani et al. [24] demonstrated an increase in the parasite burden of BALB/c macrophages infected with *L. amazonensis* when compared to macrophages from the resistant mouse strain C3H/HePas. This effect was associated with lower levels of LTB_4_ in macrophages from BALB/c mice. In agreement with this finding, macrophages from either susceptible or resistant mice treated with MK0591 (FLAP inhibitor) and U75302 (BLT1 antagonist), but with not MK571 (cysLT1 antagonist), as well as macrophages derived from 5-LO-deficient mice, exhibited decreased leishmanicidal activity. Interestingly, treatment with exogenous LTB_4_ or LTD_4_ favored parasite killing by macrophages from BALB/c mice. Supporting these *in vitro* results, susceptible and resistant mice treated with zileuton (inhibitor of 5-LO) or 5-LO-deficient mice infected with *L. amazonensis *displayed larger footpad lesions than nontreated or wild type animals [24].

The success of *Lutzomyia longipalpis*, an insect vector of the *Leishmania *ssp, at blood feeding on mammals depends on the inhibition of the immediate inflammatory response (e.g., increased vascular permeability, swelling, pain, and itching). It is well known that active substances in the saliva of hematophagous arthropods facilitate the uptake of blood by counteracting host hemostatic, inflammatory and immunological defenses [25–28]. Mixed lysates from the salivary glands of *L. longipalpis* significantly increased the cutaneous lesions and/or parasite loads in the footpads of mice infected with *L. major *or *L. braziliensis* when compared to infected animals not exposed to the saliva lysates [29, 30]. In addition, the modulation of infection by saliva was IL-4-dependent [29]. In agreement with these results, the salivary gland extract of *L. longipalpis* exhibited anti-inflammatory activities by decreasing TNF-*α* and LTB_4_ production, neutrophil numbers, and LTB_4_-induced chemotactic activity in a murine ovalbumin-induced peritonitis model [31]. In addition, IL-10 and IL-4 production was increased in this model. Taken together, these findings suggest that LTs, and particularly LTB_4_, play a role in immune response to *Leishmania* infection by promoting leishmanicidal activity and consequently, control of infection. Therefore, the modulation of LTB_4_ during infection in association with the modulation of the immune system during *Leishmania* transmission (by saliva from the insect vector) in synergism with genetic factors (susceptibility; Th2) could markedly affect *Leishmania *infection in humans.

The components derived from the saliva of the arthropod vector of malaria (e.g., *Anopheles stephensi*) have also pharmacologic effects, such as inhibition of inflammation and coagulation [32], similar to those observed in the saliva of insect vectors of *Leishmania*. In addition, these proteins also have the ability to neutralize inflammatory small molecules by rapid binding. The AnSt-D7L1 protein produced by *A. stephensi* binds cysLTs (LTC_4_, LTD_4_, and LTE_4_) but does not chemically modify them. AnSt-D7L1 effectively inhibited LTC_4_-induced ileal contraction by binding LTC_4_, thereby preventing interactions between this molecule and its appropriate cellular receptor [25]. The effects of LTC_4_ inhibition on the course of malaria infection as well as the influence in the malaria pathogenesis are not known.

In the experimental cerebral malaria model, mice infected with *Plasmodium berghei* showed increased LTB_4_ production in the serum. Interestingly, treatment with aspirin, which may direct arachidonic acid metabolism away from the cyclooxygenase (COX) pathway and toward the LO pathway [33], induced increased parasitemia and death of infected mice. This effect was associated with the overproduction of LTB_4_ in the serum [34]. In agreement with these results, children with cerebral malaria treated with salicylate demonstrated complications of severe malaria (metabolic acidosis, hypoglycemia, and death) [35]. Although IFN-*γ* plays a protective role in malaria infection, it has also been associated with the immunopathology of cerebral malaria [36, 37]. Besides playing a role in initiating the Th1 immune response mediated by dendritic cells [38], LTB_4_ is also an inducer of Th1 cytokines, such as IFN-*γ* [39]. Therefore, the overproduction of LTB_4_ after aspirin treatment in experimental and human cerebral malaria could be associated with the overproduction of IFN-*γ*. Further studies are needed to evaluate this hypothesis.

Eryptosis, or suicidal death of erythrocytes, which occurs in a wide variety of diseases including malaria [40], is characterized by cell shrinkage, membrane blebbing, and exposure of phosphatidylserine (PS) at the cell surface [41]. Like apoptotic cells, PS-exposing erythrocytes are identified by macrophages and are engulfed, degraded, and removed from the circulation [42]. Ayi et al. [43] demonstrated increased phagocytosis of mutant red blood cells infected with trophozoites of *P. falciparum*, which may represent a protective mechanism against infection. Remarkably, an *in vitro *assay demonstrated that erythrocytes were able to produce cysLTs upon energy depletion [44]. In addition, exogenous treatment with LTC_4_, but not LTB_4_, stimulated eryptosis. These effects were inhibited by cysLT1 receptor antagonists and by the 5-LO inhibitor (BW B70C) [44]. These results suggest that LTC_4_ might confer protection during the course of malaria by accelerating the clearance of infected erythrocytes. On the other hand, excessive eryptosis might favor the development of anemia; thus, LTC_4_ might have a dual effect in malaria pathogenesis.

During *T. gondii* infection, an efficient immune response is important to contain dissemination of the parasite and to prevent mortality of the host. LTC_4_, LTD_4_, and free AA were detected when murine macrophages from Swiss mice were cultured with viable *T. gondii* [45]. In contrast, when macrophages from resistant mice (BALB/c; major histocompatibility complex haplotypes H2^d^) [46] or human macrophages [47] were cultured with viable *T. gondii*, no 5-LO products were observed. Accordingly, prior incubation of human macrophages with viable *T. gondii* decreased the LTB_4_ release induced by the calcium ionophore A23187, suggesting that *T. gondii *inhibits LTB_4_ production. This effect was restored by IFN-*γ* treatment [47]. In addition, treatment with zileuton (an inhibitor of 5-LO) decreased the toxoplasmacidal activity of IFN-*γ* in human macrophages, whereas exogenous LTB_4_ promoted intracellular killing of ingested *T. gondii* in human monocytes [47]. This effect might be associated with the effect of LTB_4_ on the induction of cytotoxicity (surface membrane vesiculation, extravasation of cytoplasmic contents into a space between the intermembrane spaces and cytoplasmic vacuolization) in *T. gondii *tachyzoites [47, 48]. In agreement with these results, 5-LO-deficient mice infected with *T. gondii* displayed decreased survival as a consequence of an excessive inflammatory response characterized by elevated IL-12 and IFN-*γ* concentrations in the serum and CD4^+^ and CD8^+^ T-cell infiltration in the brain tissue and not of increased parasitic burden [49]. The increased inflammation in the absent of LTs might indicate a compensatory mechanism to control the parasite infection. Taken together, these findings suggest that the downregulation of LTs production, and particularly of LTB_4_, by *T. gondii* might be considered an evasion mechanism, as this lipid mediator can promote cytotoxicity and toxoplasmacidal activity. Thus, LTB_4_ plays an important role in toxoplasmosis.

Studies by our group and others have demonstrated reduced LT synthesis (e.g., LTB_4_) in HIV-infected subjects [14, 50]. Although the clinical manifestation of *T. gondii *infection is usually asymptomatic in immunocompetent individuals, immunocompromised individuals, such as HIV-seropositive patients, exhibit reactivation of latent tissue cysts (bradyzoites become tachyzoites) and consequent toxoplasmic encephalitis or retinochoroiditis [51, 52]. Interestingly, in agreement with these results, the LTB_4_ and LTC_4_ concentrations in the cerebrospinal fluid of HIV-1-seropositive patients with toxoplasmic encephalitis but not those of HIV-1-seropositive patients without inflammatory disease or encephalitis were below the detection limit [53]. These results support those described above and suggest that the reduced basal production of LTs in HIV-1-seropositive patients synergizes with the suppression of LTs by* T. gondii*. Moreover, this synergistic decrease in LT production might contribute to the pathogenesis of cerebral toxoplasmosis through the increased reactivation of bradyzoites from tissue cysts and the reduced control of the parasitic infection.

Protective immunity against toxoplasmosis and Chagas disease is mediated by Th1 cells, CD8^+^ T cells, and IFN-*γ* [16]. Chagas' heart disease is a severe clinical manifestation of *Trypanosoma cruzi* infection [54]. In chronic Chagas disease, cardiomyopathy is observed as an inflammatory process characterized by the infiltration of T cells and macrophages, resulting in myocarditis, fibrosis, and heart fiber damage [54]. Treatment with LT inhibitors has demonstrated beneficial effects in cardiovascular pathologies [55, 56]. T lymphocytes from patients with chronic Chagas' heart disease [57] or from chagasic mice [58] show increased contractile activity (positive inotropic and chronotropic effects) of heart (atrial) in an *in vitro* assay. Interestingly, pretreatment with lipoxygenase inhibitors (NDGA) or a cysLT receptor antagonist (FPL 55712) decreased this effect. In a separate study, LTC_4_ production was observed in the supernatants of murine atria cocultured with T lymphocytes from chagasic mice [58]. In accordance with these results, LTB_4_ induces chemotaxis of lymphocytes (CD4^+^/CD8^+^ T cells) [8, 59]. Therefore, LTs might modulate the cardiac pathology of Chagas disease by modulating the immune response profile during this infection.

LTB_4_ [60] and LTC_4_ [61] also increased the phagocytic and trypanocidal activity of murine macrophages incubated with *T. cruzi *trypomastigotes *in vitro*. In addition, LTB_4_ restored NO and TNF-*α* levels, which were decreased by an LTB_4_ receptor antagonist (CP-105,696) [62]. CP-105,696 treatment also decreased the trypanocidal activity of IFN-*γ* in murine macrophages. With the use of pharmacologic (LTB_4_ receptor antagonist and LO inhibitors) and genetic approaches (5-LO-deficient mice), researchers have demonstrated increased parasitemia in mice infected with *T. cruzi *[63–65]. In addition, the following anti-inflammatory profiles were observed in *T. cruzi *infection: (1) decreased leukocyte infiltration in the heart; (2) reduced numbers of CD4^+^, CD8^+^, and IFN-*γ*-producing cells in the heart; (3) decreased fibrosis in cardiac tissues; (4) decreased iNOS expression and NO production in the heart; (5) decreased TNF-*α* and IFN-*γ* in the heart; (6) increased IL-10 in the heart; and (7) decreased oxidative stress in erythrocytes [63–65]. The survival of 5-LO-deficient mice was greatest when the animals were infected with low number of parasites [64] when compared to animals infected with higher number of parasites [65]. Taken together, these findings suggest that LTs, and specifically LTB_4_, play important roles in the control of Chagas disease.

**Figure 2:**
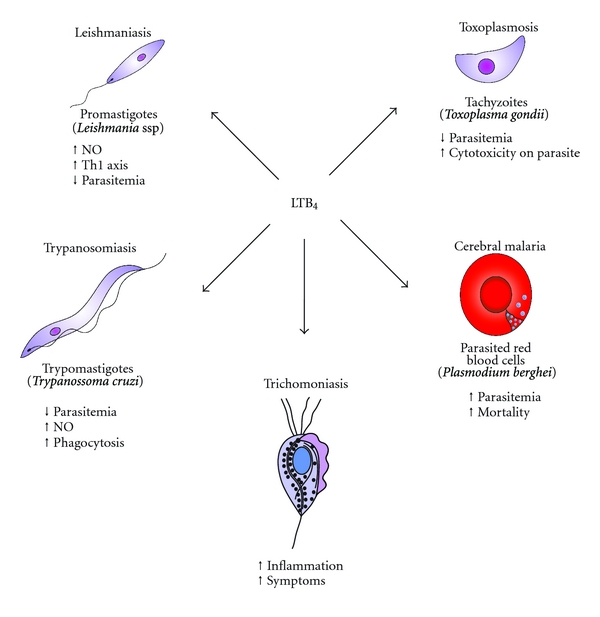
Roles of LTB_4_ on protozoan infections. LTB_4_ is involved in the control of leishmaniasis, toxoplasmosis, and trypanosomiasis. LTB_4_ is also involved in the inflammation and symptoms of trichomoniasis. The overproduction of LTB_4_, after aspirin treatment, could be associated with the exacerbated pathogenesis of cerebral malaria.

Trichomoniasis is the most common sexually transmitted disease. The supernatant of viable *T. vaginalis* induced increased LTB_4_ production in neutrophils in an IgG- and complement-(C5-) dependent manner. This effect was decreased by SC-41930 (LTB_4_ antagonist) treatment [66]. In the vaginal discharges from patients with vaginal trichomoniasis, Shaio and Lin [67] demonstrated a positive correlation between neutrophils and LTB_4_ production in symptomatic patients when compared to asymptomatic patients. These results suggest that LTB_4_ is involved in the inflammation and symptoms of trichomoniasis. The most relevant effects of LTB_4_ in protozoan infections are illustrated in Figure 2.

Entamoebiasis causes high morbidity and mortality in the developing world. Peritoneal and splenic macrophages from naïve mice incubated directly with *E. histolytica *trophozoites or with their excretory/secretory products show increased LTC_4_ production. On the other hand, peritoneal and splenic macrophages from *E. histolytica*-infected mice produced low levels of LTC_4_. Interestingly, amoebic liver abscess-derived macrophages were unable to produce LTC_4_ [68]. The downregulation of LTC_4_ by *E. histolytica* in inflammatory but not naïve macrophages might be associated with the pathogen's evasion mechanisms.

## 3. 3. Leukotrienes and Helminthic Infections

Over one-third of the human population is infected with one or more species of helminths [69, 70]. Although host immune responses attempt to control or expel the parasites, these organisms can develop evasion strategies to modulate the innate and adaptive immune responses, allowing them to survive. The most prevalent human helminthiases are caused by nematodes (e.g., *Ascaris lumbricoides*,* Strongyloides* spp., *Enterobius vermicularis,* and *Trichuris trichiura*), including filarial worms (e.g., *Brugia malayi* and *Wuchereria bancrofti*), hookworms (e.g., *Ancylostoma duodenale* and *Necator americanus*), and trematodes (*Schistosoma* spp).

Asthma and helminthiasis present similar features and are both controlled by a CD4^+^ T-cell immune response. Initial exposure of the immune system to allergic or parasitic antigens leads to the activation of a subset of T cells known as Th2 cells, which orchestrate the immune response to these exogenous antigens by secreting cytokines, including IL-4, IL-5, and IL-13 [71–74]. In addition, the accumulation of eosinophils in the blood (eosinophilia), as well as in different organs and tissues [75], is a hallmark of both diseases. Eosinophils are multifunctional cells that are involved in tissue damage as a consequence of the release of cationic proteins [76–79]. In addition, eosinophils are important sources of various inflammatory and regulatory cytokines, chemokines, and lipid mediators, such as LTs [78, 80, 81].

**Figure 3:**
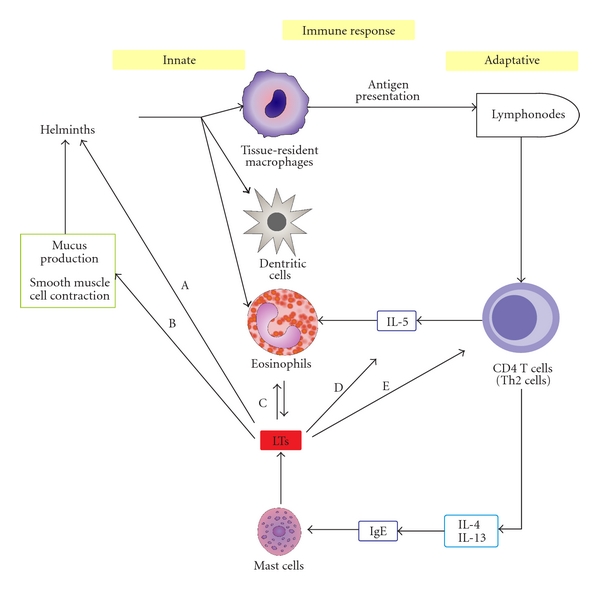
Modulation of innate and adaptive immune responses during the helminth infections. Initial exposure of the immune system to parasitic antigens leads to the activation of a subset of T cells known as Th2 cells, which orchestrate the immune response to these exogenous antigens by secreting cytokines, including IL-4, IL-5 and IL-13. The accumulation of eosinophils in the blood and in different organs and tissues as well as the degranulation of IgE-sensitized mast cells is hallmarks of helminthiasis. Eosinophils and mast cells are sources of LTs. (A) CysLTs are required for molting of the infectious larvae (e.g., *Brugia malayi *larvae). (B) CysLTs, alone or in conjunction with Th2 cytokines, cause contractility of smooth muscle cells, the permeability of epithelial cells, and the production of mucus, thereby contributing to worm expulsion. (C) LTB_4_ recruits and activates inflammatory cells such as eosinophils to favor the kill of helminths. (D) LTB_4_ regulates IL-5 production by human T lymphocytes and consequently contributes to parasite elimination. (E) LTB_4_ induces chemotaxis of CD4^+^ T cells.

During a helminth infection such as a nematode infection, most of the IgE produced binds to mast cells and basophils through their high-affinity IgE Fc receptor (Fc*ε*RI) [82, 83]. Subsequent exposure of immune cells to parasitic antigen induces the degranulation of IgE-sensitized mast cells and the release of both preformed and newly generated mediators [82]. These mediators, such as LTs, function alone or in conjunction with Th2 cytokines to increase the contractility of smooth muscle cells, the permeability of epithelial cells and the production of mucus, thereby contributing to worm expulsion [84]. The experimental gastrointestinal infection of rats with the nematode *Trichinella spiralis* demonstrated that preimmune rats (previously infected with *T. spiralis*) expelled the nematode *T. spiralis* more rapidly than nonimmune rats. This effect was associated with the increased production of LTB_4_ and LTC_4_ in the gut homogenate as well as the release of rat mast cell protease II (RMCPII) in the serum [85, 86]. LTC_4_ causes smooth muscle contraction, increases vascular permeability, and stimulates mucus hypersecretion, and LTB_4_ recruits and activates inflammatory cells such as eosinophils to favor the expulsion of helminths. Therefore, leukotrienes released from mast cells may effectively participate in protective immune responses resulting in the rapid expulsion of *T. spiralis *and possibly other helminths. The main effects of LTs, in both innate and adaptative immune responses, during the helminth infections are illustrated in Figure 3.

Parasitic worm survival in the host for longer periods depends on the ability of the parasite to evade the host immune system. The ABA-1 protein from *Ascaris lumbricoides* (human parasite) and *Ascaris suum* (pig parasite) is released by larvae and adult organisms [87, 88]. This protein binds a range of fatty acids, including LTs [89]. The interaction between ABA-1 and leukotrienes might be associated with an evasion mechanism; however, further studies are needed to evaluate the ability of this interaction to inhibit the biologic effects of LTs *in vitro* or *in vivo*.

*Brugia malayi* is a nematode (roundworm) that can cause lymphatic filariasis in humans. The infective larvae (L3) of *Brugia malayi *are transmitted to a vertebrate host by an insect vector and undergo two molts to develop into adult worms and complete the life cycle [90]. Interestingly, treatment with inhibitors of lipoxygenases (AA861) or cysLT biosynthesis (ethacrynic acid or acivicin) or with a cysLT_1_ antagonist (zafirlukast) inhibited the *Brugia malayi *L3 larvae from molting to the L4 stage without altering their survival or motility. In contrast, U-75302, an antagonist of the LTB_4_ receptor BTL_1_, failed to inhibit molting [91]. The *γ*-glutamyl transpeptidase, the enzyme that converts LTC_4_ to LTD_4_, has been cloned from *Brugia malayi* (adult worms) [92]. In another filaria that causes human infection, *Dirofilaria immitis*, the glutathione S-transferase, which can function as an LTC_4_ synthase, was found in the cytosol of adult worms [93]. These results demonstrated that a lipoxygenase pathway involved in the generation of cysLTs could be required for molting of the infectious larvae and may possibly have some role in the adult worm. *In vivo* models of infection with *B. malayi* could be used to better understand the role of cysLTs in the pathogenesis of filariasis.

It is widely known that some types of infections in immunocompromised individuals are critical in determining the severity of the disease. The immunosuppression observed in HIV-seropositive subjects has been associated with* Strongyloides* spp infections of abnormally high intensity [94]. Interestingly, reduced LT production was observed in HIV-seropositive patients [14]. In an experimental model that mimics human strongyloidiasis (mice infected with *Strongyloides venezuelensis*), an increase in the concentration of LTB_4_ but not of LTC_4_ was observed in the lung and small intestines. In addition, increased larvae recovery in the lung and/or increased worm burdens in the intestines were observed in animals treated with MK886 (a selective inhibitor of 5-lipoxygenase-activating protein (FLAP)) and in 5-LO-deficient mice than in control animals. Moreover, treatment of animals with MK886 resulted in decreases of IgG_1_ and IgE levels in serum, eosinophil numbers in the blood, peritoneal cavity and bronchoalveolar fluid volumes and IL-5 concentrations in the lung homogenate as well as increased levels of IL-12, which is involved in the Th1 response. IL-5 is the major cytokine involved in the accumulation of eosinophils in the blood during allergic inflammation and parasitic infections. This cytokine is essential for eosinophil migration from the bone marrow to the blood [72, 95] and specifically supports the terminal differentiation and proliferation of eosinophil precursors as well as the activation of mature eosinophils [96–99]. LTB_4_ regulates IL-5 production by human T lymphocytes [100] and consequently contributes to parasite elimination. These findings suggest that LTs, and specifically LTB_4_, might be necessary to control *S. stercoralis *infection. Thus, the reduced levels of LTB_4_ observed in HIV-seropositive subjects might favor opportunistic hyperinfection with* S. stercoralis*; however, further human studies are needed to evaluate this association.

*Toxocara canis *is an intestinal parasite of dogs and is the etiologic agent of toxocariasis, also known as visceral larva migrans syndrome (VLMS). Infection of both humans and animals with *T. canis *is characterized by eosinophilia in the blood and tissues, increased total serum IgE, and inflammation of the upper respiratory system [72, 95, 101–104]. During the inflammatory response, leukocyte recruitment is directly related to the expression of adhesion molecules, which allows the transmigration of these blood cells to the tissues. The integrin adhesion molecules directly contribute to this process [105]. It has been proposed that the *β*_2_ integrin Mac-1 (CD11b/CD18) and the *β*_1_ integrin VLA-4 (CD49d/CD29) adhesion molecules are the major molecules involved in cytokine- and chemokine-induced adhesion and migration of eosinophils *in vitro* [106, 107]. LTs can enhance the expression of Mac-1 on eosinophil cell surfaces [108]. In mice, *T. canis *infection causes early upregulation of Mac-1 with late changes in VLA-4 profiles on both peritoneal cavity fluid and bronchoalveolar lavage fluids, whereas MK886 treatment promoted the opposite effect. In addition, LT inhibition had a clear impact on eosinophil recruitment to tissues and on blood eosinophilia throughout the course of infection [12]. In another study, in addition to increased eosinophil numbers, the researchers showed increased numbers of mast cells in the peritoneum, lungs, and small intestines of *T. canis*-infected rats. Interestingly, these animals increased the concentration of LTB_4_ in the serum and this was correlated with mast cell and eosinophil accumulation and/or recruitment [109]. Thus, LTs might play an important role in eosinophilic inflammation during toxocariasis by inducing leukocytes recruitment and modulating the expression of adhesion molecules. 

In schistosomiasis, a granulomatous lesion is observed during chronic infection and causes a range of morbidities [110]. LTs can control parasite infection by modulating immune responses and through direct cytotoxic effects on the parasite. LTB_4_, but not cysLTs (LTC_4_ and LTD_4_), enhanced the ability of neutrophils and eosinophils to kill the schistosomula of *S. mansoni* in a complement-dependent manner [111]. The cytotoxicity of eosinophils against helminths has been associated with the expression of cellular receptors (high affinity IgE receptor, Fc*ε*RI) and adhesion molecules and with degranulation and the release of cationic proteins [112]. In an *in vitro *assay, IgE-coated schistosomula induced eosinophil adherence, resulting in the death of the parasites. In addition, the release of LTC_4_ was observed during this interaction [113]. In agreement with this finding, schistosomula can produce LTB_4_ and LTC_4_ [114]. The function of LTs in schistosomula is not known; however, their production might accelerate parasite elimination and/or modulate the pathogenesis of schistosomiasis.

Schistosome cercariae enter mammalian hosts via a percutaneous route [115]. In addition to the proteolytic enzymes produced by cercariae, host-derived skin essential fatty acids and LTs including LTB_4_ also play important roles in the penetration of the skin by the parasite. In an *in vitro* assay, increased penetration rates were correlated with increased LTs levels. In addition, penetration was reduced upon treatment with a 5-LO inhibitor [116, 117].

**Figure 4:**
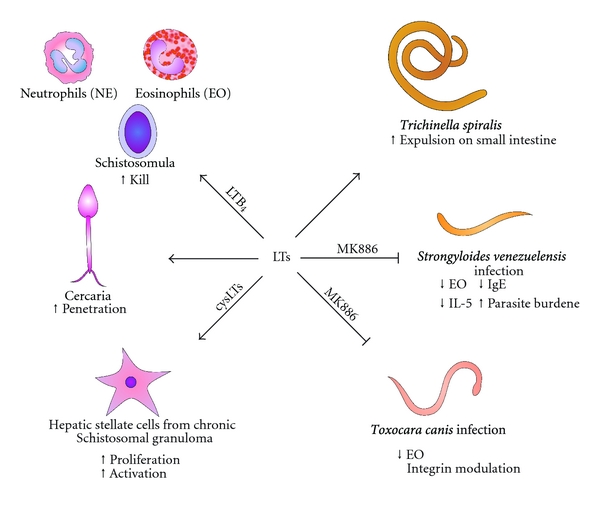
An overview of main effects of LTs in helminth infections. LTs participate of expulsion of *T. spiralis* in the intestine. The inhibition of LTs resulted in increases of parasitemia and decreases IgE levels, eosinophil numbers and IL-5 concentrations in mice infected with *S. venezuelensis*. LT inhibition also reduced the eosinophil recruitment to tissues and on blood eosinophilia in experimental *T. canis* infection. LTB_4_ enhanced the ability of neutrophils and eosinophils to kill the schistosomula of *S. mansoni*. LTs play important roles in the penetration of the skin by the schistosome cercariae. CysLTs are involved in the proliferation and activation of hepatic stellate cells from *S. mansoni* granulomas.

Hepatic granulomatous inflammation is observed during schistosomal infection of both humans and mice [110]. Th2 cell-associated cytokines modulate the development of schistosome egg-induced granulomas. Hepatic stellate cells (HSCs) are involved in liver remodeling due to collagen production and deposition of extracellular matrix as a consequence of proliferative and fibrogenic phenotypes induced by several mediators (cytokines, lipid peroxide, and others) [118]. mRNA for 5-LO, FLAP and LTC_4_-synthase and 5-LO expression was observed in HSCs from schistosomal granulomas of *S. mansoni*-infected mice [119]. Consequently, these cells produced cysLTs, but not LTB_4_, and the production of cysLTs was increased upon treatment with transforming growth factor beta (TGF-*β*, a fibrogenic cytokine). The proliferation induced by TGF-*β* in HSCs from schistosomal granulomas of *S. mansoni*-infected 5-LO-deficient mice or wild type mice treated with zileuton (5-LO inhibitor) was reduced [119]. In addition, LTC_4_ induced TGF-*β* production [120], suggesting a synergic effect in schistosomal granulomas. In another study, dipeptidases were isolated from extracts of hepatic granulomas of mice infected with *S. mansoni*; these enzymes increased the hydrolysis of LTD_4_ to LTE_4_ [121], potentially accelerating the metabolism of LTs and decreasing their effects on liver remodeling. Moreover, LTB_4_ and LTC_4_ are produced by schistosomula and adult females, while males produced only LTB_4_ [113]. Together, these results suggest that cysLT inhibition might influence liver remodeling in *S. mansoni* infection. In this way, CysLT_1_ antagonists (such as montelukast, zafirlukast, and pranlukast) [4, 122, 123], which are currently used in asthma treatment, could be evaluated for their effects on schistosomal granuloma remodeling in experimental or human schistosomiasis. The main roles of LTs during the helminth infection are illustrated in Figure 4.

Similar to schistosomiasis, fasciolosis causes liver alterations, which can range from fibrosis to cirrhosis. Fasciolosis is considered both a human health concern and a veterinary problem (zoonoses) [124]. During the course of *F. hepatica* infection in sheep, a reduction in serum LTB_4_ was observed when compared to control animals. Interestingly, LTB_4_ was produced in both the culture supernatant and the homogenate of *F. hepatica* adult parasites recovered from the bile duct 20 weeks after infection [125]. Moreover, recruitment of leukocytes consisting mainly of eosinophils, macrophages, and lymphocytes was observed in the livers of goats infected with *F. hepatica* [126]. In this way, LTB_4_ produced by host inflammation in synergy with that produced by the parasite could contribute to liver alterations and consequent pathology.

## 4. Conclusion

LTs are associated with the control of helminth and protozoan infections through their ability to modulate inflammatory processes and/or to promote direct cytotoxicity of protozoans. In addition, LTs may also be associated with exacerbated pathogenesis in protozoan diseases, such as cerebral malaria, and helminthic diseases, such as schistosomal granulomas. Interestingly, some helminths (*B. malayi*) might use the LTs to complete their development to adult worms. In addition, other parasites produce LTs (*S. mansoni* and *F. hepatica*) or produce enzymes involved in LT biosynthesis (*Dirofilaria immitis*). Taken together, these findings demonstrate that LTs play significant roles in protozoan and helminth infections.

## List of Abbreviations

5-LO: 5-Lipoxygenase
AA: Arachidonic acid
cysLTs: Cysteinyl leukotrienes
FLAP: Membrane-bound 5-lipoxygenase-activating protein
HSCs: Hepatic stellate cells
IFN-*γ*: Interferon-gamma
Ig: Immunoglobulin
IL: Interleukin
LTs: Leukotrienes
NO: Nitric oxide
PS: Phosphatidylserine
TGF-*β*: Transforming growth factor-beta
Th: T helper
TNF-*α*: Tumor necrosis factor-alpha
VLMS: Visceral larva migrans syndrome.
